# Hematological Abnormalities in Cirrhosis: A Narrative Review

**DOI:** 10.7759/cureus.39239

**Published:** 2023-05-19

**Authors:** Elvina C Lingas

**Affiliations:** 1 Internal Medicine, Presbyterian Hospital, Albuquerque, USA

**Keywords:** hematological abnormalities, etiology and pathogenesis, liver cirrhosis . fibrosis, thrombocytopenia, s: anemia

## Abstract

Liver cirrhosis remains a major public health issue. Liver fibrosis leading to cirrhosis is the terminal stage of various chronic liver diseases. Inflammatory cytokines are involved in the pathogenesis. Patients with cirrhosis often have hematological abnormalities, such as anemia and thrombocytopenia, which have multifactorial etiologies. Anemia in cirrhosis could be related to bleeding leading to iron deficiency anemia or other nutritional anemia such as vitamin B12 and folate deficiency. The pathophysiology of thrombocytopenia in liver cirrhosis has been postulated to range from splenic sequestration to bone marrow suppression from toxic agents, such as alcohol. It often complicates management due to the risk of bleeding with severely low platelets. This review aimed to highlight pathogenesis of liver cirrhosis, hematological abnormalities in liver cirrhosis, and their clinical significance.

## Introduction and background

**Figure 1:**
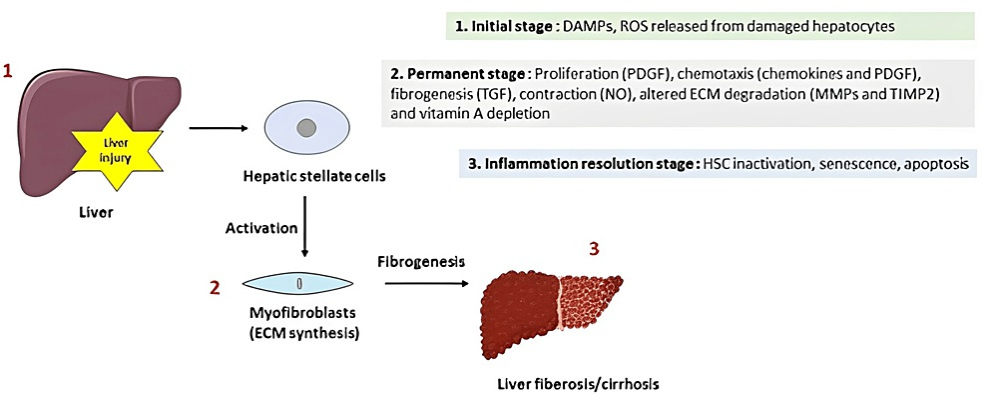
Schematic representation of pathogenesis of liver fibrosis and cirrhosis. Liver injury primarily activates hepatic stellate cells (HSCs) known to be involved in collagen synthesis (ECM). Activation and proliferation of HSCs contribute to liver fibrosis and later liver cirrhosis. This occurs in three stages - (1) initial stage in which liver injury causes release of danger-associated molecular proteins (DAMPs), reactive oxygen species (ROS), etc.; (2) this leads to the activation of HSCs, which undergo several cellular changes, such as proliferation, contraction, chemotaxis, fibrogenesis, ECM alteration, etc.; (3) at the end HSCs inactivation, senescence, and apoptosis take place. Death of hepatocytes by injury invites Kupffer cells to secrete more cytokines that transform HSCs into functionally different cells (myofibroblasts) that secrete excessive amounts of collagens known to cause fibrosis. ECM: extracellular matrix The image is created by the author (Lingas EC) of this study.

Cirrhosis is described as scarring of the liver causing scar tissues or fibrotic tissues to eventually replace healthy liver tissues that develop over time [1]. Increasingly fibrotic tissues are causing liver dysfunction although often asymptomatic in the early stages. Liver cirrhosis remains one of the top 10 leading causes of death [2,3]. Approximately 160 million people in the world suffered from cirrhosis, and approximately 0.8 million patients with cirrhosis die every year [4,5]. Some studies have shown that hepatitis C virus (HCV) and alcohol are the major causes of cirrhosis in the United States, most European countries, and Japan. Cirrhosis caused by hepatitis B virus (HBV) mainly occurred in Asian-Pacific and African countries [6]. Majority of the liver diseases destroy healthy liver cells, leading to cell death and inflammation (Figure 1). Fibrosis occurs as a mechanism of cellular repair and ongoing fibrosis results in non-functioning liver cells leading to further cellular apoptosis. Late-stage cirrhosis has a myriad of life-threatening complications including hematological abnormalities.

## Review

Incidence

In the United States liver cirrhosis affects about one in 200 adults from the age of 45-54 years. Cirrhosis causes about 26,000 deaths each year in the United States and is the seventh leading cause of death in the United States among adults 25-64 years of age. A higher incidence of liver cirrhosis is shown in men [7].

Risk factors

**Figure 2:**
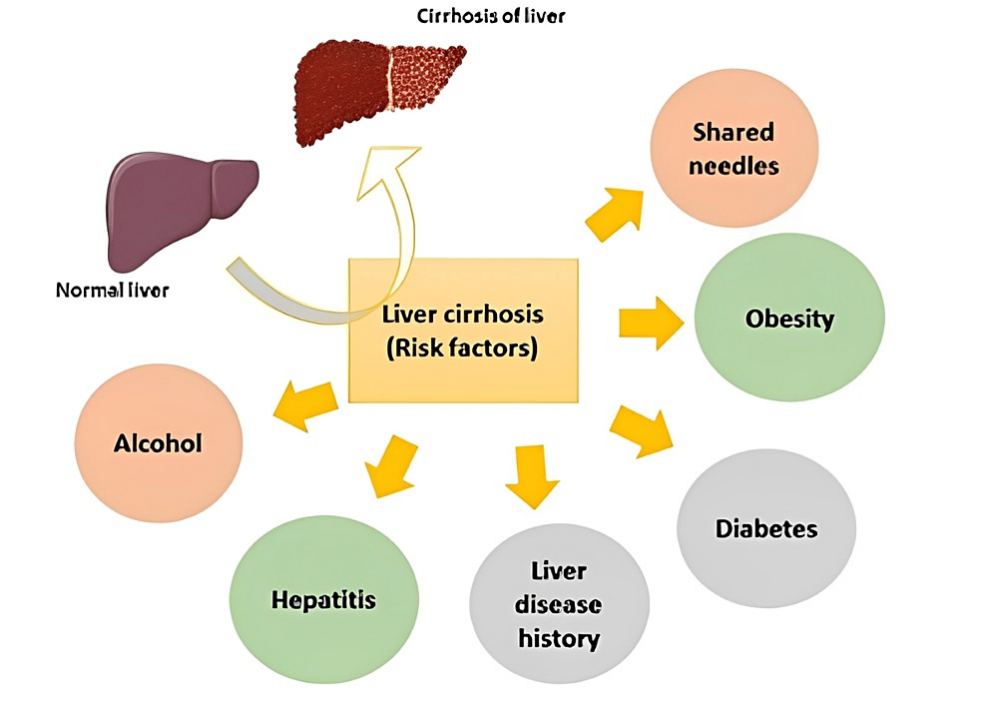
Schematic representation of risk factors involved in liver cirrhosis. Use of alcohol for longer time, chronic hepatitis B and C virus infection, history of liver disease, diabetes, obesity, and drugs injected through shared needles cause liver cirrhosis that affects the normal functioning of hepatocytes. The image is created by the author (Lingas EC) of this study.

Possible etiologies of liver cirrhosis are shown in Figure 2. A 2020 study showed that the most common causes were alcohol use disorder (50.5%), cryptogenic cirrhosis (14.5%), viral hepatitis C (13.4%), and non-alcoholic fatty liver disease (5.7%). Hypertension, obesity, and type 2 diabetes mellitus (DM) are the most common co-morbidities [7].

Pathogenesis

Various types of cells, inflammatory cytokines as well as mRNAs are found to trigger the progression from fibrosis to liver cirrhosis [8]. Liver cirrhosis stage includes degeneration and necrosis of hepatocytes, loss of liver function and fibrotic tissues as well as regenerative nodules that eventually replace liver parenchyma [9-11]. Fibrosis, the precursor of cirrhosis is a key pathological process in the progress of chronic liver diseases to cirrhosis [12,13]. Current studies are still lacking a concise understanding of the exact pathogenesis of cirrhosis.

Cell Types Involved in the Pathogenesis of Liver Cirrhosis

The following cell types form liver cirrhosis: hepatocyte and non-hepatocyte cells. Hepatic sinusoidal cell walls comprise non-parenchymal cells which are liver sinusoidal endothelial cells (LECs), Kupffer cells (KCs), and hepatic stellate cells (HSCs). Both cell types favor the initiation and progression of liver fibrosis and cirrhosis (Table 1).

**Table 1:** Liver cell type, function, and role in liver cirrhosis. MMP: matrix metalloproteinases; TIMP: tissue inhibitors metalloproteinases

Cell type	Function	Role in liver cirrhosis
Hepatic stellate cells (HSCs)	Liver-specific mesenchymal cells that play vital roles in liver physiology, vitamin A storage	HSC activation initiates hepatic fibrosis and later liver cirrhosis
Liver sinusoidal endothelial cells (LSECs)	Constitute the sinusoidal wall	Secrete cytokine IL-33 to activate HSCs and promote fibrosis. It can also revert activated HSCs and inhibit fibrosis in liver
Kupffer cells (KCs)	Specialized macrophages (innate immunity)	Destroy hepatocytes by producing harmful soluble mediators upon injury
Hepatocytes	Primary liver parenchymal cells	Main producer of MMP-2, MMP-3 and MMP-13 and tissue inhibitors such as TIMP-1 and TIMP-2

HSCs

HSCs are fat-storing cells, also known as lipocytes, Ito cells, perisinusoidal cells, or vitamin A-rich cells, that reside in the normal liver and their main purpose is vitamin A and retinoid storage [14-16]. Inflammatory cytokines, such as PDGF, TGF, TNF-α, and IL-1, activate HSC and initiate hepatic fibrosis leading to collagen deposition [16,17]; that ultimately leads to fibrosis [18-20].

LSECs

Liver sinusoidal endothelial cells (LSECs) constitute sinusoidal wall which is part of endothelium [12,21,22]. Both animal and human studies showed that LSECs can secrete the cytokine IL-33 that promotes fibrosis [23]. Defenestration and capillarization of LSECs disturb substrate exchange and greatly contribute to liver dysfunction leading to cirrhosis [24].

KCs

KCs, which are also known as Browicz-Kupffer cells and stellate macrophages, are a specialized type of macrophages that comprise the reticuloendothelial system (RES) [25]. Animal studies suggest that KCs play a role in various liver diseases’ pathogenesis [26,27]. Injurious factors, such as viral infection, alcohol use, dietary factors, such as high-fat diet, and iron accumulation may trigger KCs. Activated KCs act as antigen-presenting cells during viral infection, leading to the production of harmful substances [26]. KC-mediated hepatic inflammation aggravates liver injury and fibrosis [28,29].

Hepatocytes

Hepatocytes are the primary liver parenchymal cells and are affected by hepatotoxic substances, such as hepatitis viruses, alcohol, and bile acids metabolites [30]. Hepatocytes produce matrix metalloproteinases (MMP-2, MMP-3, and MMP-13) and tissue inhibitor metalloproteinases (TIMP-1 and TIMP-2), all are important in liver cirrhosis pathogenesis [31]. Recent studies discovered that hepatocyte telomere shortening and senescence may lead to progressive scarring of liver tissues leading to fibrosis and ultimately cirrhosis [32].

Hematology abnormality in cirrhosis

Hematological indices (HI) or abnormalities in hematological parameters are found in cirrhotic patients (Table 2). Studies have shown that around 6-77% of cirrhotic patients often have abnormal HI which includes anemia, thrombocytopenia, and leukopenia [33,34]. Most studies have evaluated HI in a cross-sectional manner, and the sequential development of anemia, leukopenia, and thrombocytopenia is not known. The etiology is multifactorial, including splenic sequestration, bone marrow suppression, and disturbance in the balance of hematopoietic stimulating factors [35]. Abnormalities in HIs are associated with an increased risk of bleeding and infection. The liver has a pivotal role in the maintenance of homeostasis. Any disturbance in this dynamic process results in many aberrations, which include hematological manifestations such as anemia, leukopenia, and thrombocytopenia [36].

**Table 2:** Types of hematologic abnormalities in cirrhosis and its causes.

Hematologic abnormalities	Causes
Thrombocytopenia	(1) Portal hypertension-induced splenic sequestration, (2) bone marrow suppression toxins, such as hepatitis C and alcohol, (3) consumptive coagulopathy (e.g., low-grade hemolysis) and increased blood loss (e.g., hemorrhage)
Leukopenia	(1) Portal hypertension-induced splenic and splanchnic sequestration, (2) disturbances in granulocyte-colony stimulating factor and granulocyte macrophage-colony stimulating factor, (3) bone marrow suppression mediated by toxins (e.g., alcohol, hepatitis B and C)
Anemia	Lower production of healthy red blood cells, deficiency of vitamin B12, folate, and iron

Anemia

Anemia is a common complication present in most patients with liver cirrhosis but is often missed at the early stage. The liver is a storage site of vitamins and minerals such as B12, folic acid, vitamin E, iron, and copper. The liver also is the major organ that produces hepcidin, an iron-regulating hormone, which is expressed in a high inflammatory state as well as iron-rich state, which blocks the absorption of iron by enterocytes [37]. Anemia is a condition that occurs due to lower production of healthy red blood cells or lower levels of hemoglobin and often is caused by iron deficiency, or B12 and folate deficiency [38]. The most common type of anemia observed in cirrhosis patients is normocytic normochromic anemia which is attributed to a chronic inflammatory state [39]. Patients who were found to have anemia with cirrhosis seem to have a higher hospital mortality rate. Patients with poorer Child-Pugh scores and higher Model for End-stage Liver Disease (MELD) scores seem to have more severe forms of anemia [40]. Anemia also has been shown to have a role in hepatorenal syndrome. It is crucial to conduct more studies specifically studying the clinical significance of hematological abnormalities in cirrhosis, such as anemia [40].

Pathogenesis

Anemia in cirrhosis is a complex entity. Patients with cirrhosis often have chronic bleeding leading to iron deficiency, they also often have nutritional deficiencies such as vitamin B12 and folate deficiency. Hepcidin as previously mentioned is an iron-regulating hormone, which is produced by the liver to maintain iron homeostasis. Dietary iron absorption is blocked by hepcidin when iron levels in plasma and iron storage exceed maximum capacity. In contrast to this, hepcidin production is suppressed in iron deficiency which increases dietary iron absorption. In chronic inflammation of the liver (cirrhosis), hepcidin production is interceded by interleukin-6 (IL-6) dependent and IL-6 independent pathways. It is important to make a note that in a condition like cirrhosis, hepcidin levels are not downregulated even in low levels of iron in plasma. The pathophysiological role of hepcidin in the development of anemia in cirrhosis is best explained by suppressor of mothers against decapentaplegic (SMAD) signaling pathway, wherein, IL-6 an inflammatory mediator binds to the IL-6 receptor and activates the Janus kinases signal transducer and activator of transcription protein-3 (JAK-STAT-3) pathway. STAT-3 thereby causes an elevation in levels of hepcidin [40]. Another imperative pathway involves the binding of transferrin (Tf) to transferrin receptor-1 (Tfr-1). This binding breaks down transferrin receptor-1-human homeostatic iron regulator (Tfr-1 HFE) complex. Interaction between HFE and Tfr-2 is shown to increase bone morphogenetic protein (BMP)6-mediated phosphorylation of SMAD1/5/8, which increases hepcidin expression by further recruiting SMAD4 [40]. Patients with cirrhosis also may have spontaneous hemolysis and the presence of hemolytic cells such as spur cells carries a poor prognosis. It is postulated that severe liver dysfunction may lead to abnormal lipid and protein production that lead to dysmorphic RBCs and increased hemolysis [41].

Diagnosis and Treatment

There are initial tests to evaluate anemia that have been widely accepted, such as Hb level, platelet count, RBC indices, white blood count (WBC), differential cell count (DLC), mean corpuscular volume (MCV), absolute reticulocyte count, serum iron studies, transferrin saturation (TSAT), serum ferritin, and hepcidin [40].

A complete blood count (CBC) is the primary diagnostic test for screening for anemia. CBC, however, has low sensitivity and specificity and has limitations when used alone. In addition to Hb, erythrocyte parameters like MCV, mean corpuscular hemoglobin (MCH), and red cell distribution width (RDW) are measured in combination [37].

The supply of iron is imperative for erythropoiesis. Transferrin saturation (Tsat) may be used to diagnose iron deficiency anemia. Levels of Tsat lower than 16% are indicative of an insufficient supply of iron. Guidelines suggest initiating treatment at a cut-off of <20%. Levels of Tsat can fluctuate with transferrin, elevated levels of transferrin in inflammations may lower the Tsat. In contrast, suppressed transferrin synthesis due to malnourishment and chronic disease might raise the Tsat, which makes it difficult to interpret.

Ferritin levels are proven to be superior to other diagnostic measures, with levels ≤12 µg being diagnostic of iron deficiency. However, ferritin is an acute-phase protein affected by iron status and inclusive of acute or chronic inflammation, malignancies, and liver disease making it a challenging test. Soluble transferrin receptor or sTfR additionally may also be used. sTfR ties in the fact that the concentration of membrane of erythroblasts in the bone marrow increases in the state of ID. This test has high sensitivity but low specificity, although, specificity is improved significantly when the sTfR-ferritin index was used which makes it a more reliable indicator of iron deficiency [37]. Treatment of anemia in cirrhosis depends on the etiology. For symptomatic anemia related to variceal bleeding in cirrhosis transfusion is given for Hb less than 7 g/dL and maintaining the level between 7 and 9 g/dL has been shown to improve survival in Child-Pugh A and B cirrhosis as well as decrease rebleeding risk [42-44]. Iron replacement is also used to treat iron deficiency leading to anemia in cirrhosis. Oral iron replacement is convenient but often associated with gastrointestinal side effects. Parenteral iron is also often given, especially in inpatient settings [45]. Iron replacement specifically in anemia in cirrhosis is not yet well studied, although a study performed by Rashidi-Alavijeh et al. showed that iron replacement increases hemoglobin levels and is significantly associated with increased survival [46].

Thrombocytopenia

Thrombocytopenia is a common hematological abnormality observed in liver disease. It increases the risk of bleeding and can limit planned surgical/diagnostic procedures due to this. Thrombocytopenia affects approximately more than half of patients diagnosed with cirrhosis. It is considered diagnostic if a platelet count of less than 150,000/μL, with mild thrombocytopenia is defined as having a platelet count of 100,000-150,000/μL, moderate thrombocytopenia as having a platelet count of 50,000-100,000/μL, and severe thrombocytopenia as less than 50,000/μL [35]. Advanced liver disease patients often have thrombocytopenia and some studies have suggested thrombocytopenia as an independent predictor of mortality [33,47]. There are many hypotheses regarding thrombocytopenia mechanism in cirrhosis.

Pathogenesis

Platelet production is associated with thrombopoietin (TPO). TPO is mainly produced by the liver, kidney, muscle as well as bone marrow stromal cells, and its synthesis is mainly dependent on hepatic function. TPO is bound to c-Mpl receptor located on megakaryocytes and regulates differentiation into platelets [48]. It is postulated that circulating TPO level is associated with cirrhosis stages as well as the severity of thrombocytopenia. Increased fibrosis leads to reduced levels of circulating TPO, thus the worsening of thrombocytopenia [49]. Multiple factors, such as splenic sequestration, decreased hematopoietic growth factor and thrombopoietin, and bone marrow suppression from viral infections such as hepatitis C as well as anti-cancer agents, and antiviral treatment with interferon-based therapy, all may contribute to the development of thrombocytopenia in cirrhotic patients [50].

Cirrhosis patients have increased platelet destruction as well. Immune-mediated destruction plays a great role in platelet destruction, specifically shown in autoimmune liver diseases and chronic hepatitis C virus (HCV). Sepsis may also contribute significantly to platelet destruction. Patients with cirrhosis have a higher risk of developing sepsis and multiple inflammatory cytokines, such as tumor necrosis factor, have been shown to cause platelet destruction [50]. Pulmonary hypertension and pulmonary emboli are also associated with platelet consumption and it is important to diagnose these conditions in cirrhotic patients [51].

Diagnosis and Treatment

Thrombocytopenia carries an increased bleeding risk, especially in cirrhotic patients who often require procedures. Paracentesis and esophagogastroduodenoscopy usually have lower bleeding risk, however, other procedures, such as liver biopsies, chemoembolizations, transjugular intrahepatic portosystemic shunts (TIPSs), and biliary procedures do carry a higher risk of bleeding [52]. In a study of bleeding complications after liver biopsy in patients with hepatitis C cirrhosis, thrombocytopenia caused 11% of scheduled biopsies to be missed, and severe thrombocytopenia with a platelet count of less than 60,000/μL showed significant bleeding risk [53].

Standard treatments of thrombocytopenia in cirrhotic patients include platelet transfusions, surgical splenectomy, and interventional splenic artery embolization; all had been shown to improve thrombocytopenia in recent studies. Improvement in platelet levels may reduce the need for platelet transfusions and allow the use of interferon antiviral therapy in cirrhotic patients [54].

Few studies showed the efficacy of TIPS, however, the mechanism of how it improves thrombocytopenia is still uncertain [55]. TPO receptor agonists have been used recently, especially in patients who are considered poor surgical candidates. The mechanism of these medications is related to the human TPO receptor (c-Mpl) and is shown to promote the proliferation of megakaryocytes, therefore, improving platelet count. In 2008 eltrombopag was approved by FDA for the treatment of idiopathic thrombocytopenic purpura (ITP).

Afdhal et al. showed eltrombopag reduced the necessity for platelet transfusions in patients with chronic liver disease who needed elective invasive procedures, however, it increased portal vein thrombosis risk and therefore is not recommended for patients with chronic liver disease who need elective procedures [56]. Avatrombopag and lusutrombopag, however, were approved by FDA in 2018 for cirrhotic patients with thrombocytopenia undergoing procedures. Both have been shown to lower pre-procedural platelet transfusions' frequency as well as post-procedure bleeding [57,58]. In addition, TPO receptor agonists showed reduced costs when compared with the costs of multiple platelet transfusions [59].

Prognosis

Further studies are required to explore the association of morbidity and mortality with thrombocytopenia and the role of TPO receptor agonists play in increasing survival in patients with chronic liver disease and cirrhosis. Some studies have suggested thrombocytopenia as an independent predictor of mortality [33,47]. Recent evidence has suggested hypersplenism’s role in increasing morbidity and mortality in cirrhosis [60]. A 2020 study by Scheiner et al. showed that aside from higher prevalence of anemia in patients with advanced liver disease, patients with severe anemia have higher rate of hospitalization and decompensation, which could translate to higher mortality and worse survival [61]. Increasing evidence suggested that anemia could be used as an independent predictor of acute decompensation of cirrhosis aside from MELD score [62]. Overall we are seeing increasing evidence that hematological abnormality in cirrhosis is clinically significant and may be further studied to aid in determining prognosis and survival.

## Conclusions

Cirrhosis remains a major public health problem. Several studies have evaluated the mechanisms responsible for regulation of primary hemostasis, coagulation, and fibrinolysis, which are severely impaired in cirrhosis. The etiology of cirrhosis is multifactorial and the mechanisms underlying pathogenesis of cirrhosis are still unclear. Cirrhosis often has multiple hematologic abnormalities, such as anemia and thrombocytopenia. Anemia in cirrhosis is usually related to hemorrhage, iron deficiency, and nutrition; its pathogenesis is complex. The major mechanisms of thrombocytopenia in liver cirrhosis include platelet sequestration and decreased TPO production. For thrombocytopenia caused by the latter, TPO agonists and targeted agents’ non-invasive nature may have increased roles for cirrhotic patients in the future. Patients with abnormal hematologic indices tend to have poorer prognosis and increased mortality.

## References

[1] Ye F, Zhai M, Long J (2022). The burden of liver cirrhosis in mortality: results from the global burden of disease study. Front Public Health.

[2] Tsai TY, Hung TH, Livneh H, Lin IH, Lu MC, Yeh CC (2018). Chinese herbal medicine therapy and the risk of mortality for chronic hepatitis B patients with concurrent liver cirrhosis: a nationwide population-based cohort study. Oncotarget.

[3] Corrao G, Ferrari P, Zambon A, Torchio P, Aricò S, Decarli A (1997). Trends of liver cirrhosis mortality in Europe, 1970-1989: age-period-cohort analysis and changing alcohol consumption. Int J Epidemiol.

[4] Liu Z, Jiang Y, Yuan H (2019). The trends in incidence of primary liver cancer caused by specific etiologies: results from the Global Burden of Disease Study 2016 and implications for liver cancer prevention. J Hepatol.

[5] Blachier M, Leleu H, Peck-Radosavljevic M, Valla DC, Roudot-Thoraval F (2013). The burden of liver disease in Europe: a review of available epidemiological data. J Hepatol.

[6] Michitaka K, Nishiguchi S, Aoyagi Y, Hiasa Y, Tokumoto Y, Onji M (2010). Etiology of liver cirrhosis in Japan: a nationwide survey. J Gastroenterol.

[7] Vaz J, Eriksson B, Strömberg U, Buchebner D, Midlöv P (2020). Incidence, aetiology and related comorbidities of cirrhosis: a Swedish population-based cohort study. BMC Gastroenterol.

[8] Zhou WC, Zhang QB, Qiao L (2014). Pathogenesis of liver cirrhosis. World J Gastroenterol.

[9] Anthony PP, Ishak KG, Nayak NC, Poulsen HE, Scheuer PJ, Sobin LH (1978). The morphology of cirrhosis. Recommendations on definition, nomenclature, and classification by a working group sponsored by the World Health Organization. J Clin Pathol.

[10] Anthony PP, Ishak KG, Nayak NC, Poulsen HE, Scheuer PJ, Sobin LH (1977). The morphology of cirrhosis: definition, nomenclature, and classification. Bull World Health Organ.

[11] Ferrell L (2000). Liver pathology: cirrhosis, hepatitis, and primary liver tumors. Update and diagnostic problems. Mod Pathol.

[12] Braet F, Wisse E (2002). Structural and functional aspects of liver sinusoidal endothelial cell fenestrae: a review. Comp Hepatol.

[13] Asrani SK, Larson JJ, Yawn B, Therneau TM, Kim WR (2013). Underestimation of liver-related mortality in the United States. Gastroenterology.

[14] Elsharkawy AM, Oakley F, Mann DA (2005). The role and regulation of hepatic stellate cell apoptosis in reversal of liver fibrosis. Apoptosis.

[15] Friedman SL (1993). The cellular basis of hepatic fibrosis - mechanisms and treatment strategies. N Engl J Med.

[16] Lakner AM, Steuerwald NM, Walling TL (2012). Inhibitory effects of microRNA 19b in hepatic stellate cell-mediated fibrogenesis. Hepatology.

[17] Oakley F, Meso M, Iredale JP (2005). Inhibition of inhibitor of kappa B kinases stimulates hepatic stellate cell apoptosis and accelerated recovery from rat liver fibrosis. Gastroenterology.

[18] Safadi R, Friedman SL (2002). Hepatic fibrosis - role of hepatic stellate cell activation. MedGenMed.

[19] Gressner AM, Weiskirchen R (2006). Modern pathogenetic concepts of liver fibrosis suggest stellate cells and TGF-beta as major players and therapeutic targets. J Cell Mol Med.

[20] He Y, Huang C, Zhang SP, Sun X, Long XR, Li J (2012). The potential of microRNAs in liver fibrosis. Cell Signal.

[21] Mori T, Okanoue T, Sawa Y, Hori N, Ohta M, Kagawa K (1993). Defenestration of the sinusoidal endothelial cell in a rat model of cirrhosis. Hepatology.

[22] Straub AC, Stolz DB, Ross MA, Hernández-Zavala A, Soucy NV, Klei LR, Barchowsky A (2007). Arsenic stimulates sinusoidal endothelial cell capillarization and vessel remodeling in mouse liver. Hepatology.

[23] Marvie P, Lisbonne M, L'helgoualc'h A (2010). Interleukin-33 overexpression is associated with liver fibrosis in mice and humans. J Cell Mol Med.

[24] Yokomori H, Oda M, Yoshimura K, Hibi T (2012). Recent advances in liver sinusoidal endothelial ultrastructure and fine structure immunocytochemistry. Micron.

[25] Kmieć Z Cooperation of liver cells in the synthesis and degradation of eicosanoids. Cooperation of Liver Cells in Health and Disease. Advances in Anatomy Embryology and Cell Biology.

[26] Kolios G, Valatas V, Kouroumalis E (2006). Role of Kupffer cells in the pathogenesis of liver disease. World J Gastroenterol.

[27] Noda T, Mimura H, Orita K (1990). Assessment of Kupffer cell function in rats with chronic liver injury caused by CCl4. Hepatogastroenterology.

[28] López-Navarrete G, Ramos-Martínez E, Suárez-Álvarez K (2011). Th2-associated alternative Kupffer cell activation promotes liver fibrosis without inducing local inflammation. Int J Biol Sci.

[29] Vollmar B, Siegmund S, Richter S, Menger MD (1999). Microvascular consequences of Kupffer cell modulation in rat liver fibrogenesis. J Pathol.

[30] Bataller R, Brenner DA (2005). Liver fibrosis. J Clin Invest.

[31] Garcíade León Mdel C, Montfort I, Tello Montes E (2006). Hepatocyte production of modulators of extracellular liver matrix in normal and cirrhotic rat liver. Exp Mol Pathol.

[32] Wiemann SU, Satyanarayana A, Tsahuridu M (2002). Hepatocyte telomere shortening and senescence are general markers of human liver cirrhosis. FASEB J.

[33] Qamar AA, Grace ND, Groszmann RJ (2009). Incidence, prevalence, and clinical significance of abnormal hematologic indices in compensated cirrhosis. Clin Gastroenterol Hepatol.

[34] Qamar AA, Grace ND, Groszmann RJ (2008). Platelet count is not a predictor of the presence or development of gastroesophageal varices in cirrhosis. Hepatology.

[35] Peck-Radosavljevic M (2017). Thrombocytopenia in chronic liver disease. Liver Int.

[36] Marks PW (2013). Hematologic manifestations of liver disease. Semin Hematol.

[37] Gkamprela E, Deutsch M, Pectasides D (2017). Iron deficiency anemia in chronic liver disease: etiopathogenesis, diagnosis and treatment. Ann Gastroenterol.

[38] Singh S, Manrai M, Parvathi VS, Kumar D, Srivastava S, Pathak B (2020). Association of liver cirrhosis severity with anemia: does it matter?. Ann Gastroenterol.

[39] Dawidowski J, Pietrzak A (2022). Rare causes of anemia in liver diseases. Adv Clin Exp Med.

[40] Manrai M, Dawra S, Kapoor R, Srivastava S, Singh A (2022). Anemia in cirrhosis: an underestimated entity. World J Clin Cases.

[41] Barron K, Ramadas P, Grewal US (2022). Hemolytic anemia in a patient with cirrhosis: hiding in the smear!. Eur J Intern Med.

[42] D'Amico G, Pagliaro L, Bosch J (1999). Pharmacological treatment of portal hypertension: an evidence-based approach. Semin Liver Dis.

[43] de Franchis R (2015). Expanding consensus in portal hypertension: report of the Baveno VI Consensus Workshop: stratifying risk and individualizing care for portal hypertension. J Hepatol.

[44] Handel J, Lang E (2015). Transfusion strategy for acute upper gastrointestinal bleeding. CJEM.

[45] Goddard AF, James MW, McIntyre AS, Scott BB (2011). Guidelines for the management of iron deficiency anaemia. Gut.

[46] Rashidi-Alavijeh J, Nuruzade N, Frey A (2023). Implications of anaemia and response to anaemia treatment on outcomes in patients with cirrhosis. JHEP Rep.

[47] Bleibel W, Caldwell SH, Curry MP, Northup PG (2013). Peripheral platelet count correlates with liver atrophy and predicts long-term mortality on the liver transplant waiting list. Transpl Int.

[48] Kaushansky K (2006). Lineage-specific hematopoietic growth factors. N Engl J Med.

[49] Mitchell O, Feldman DM, Diakow M, Sigal SH (2016). The pathophysiology of thrombocytopenia in chronic liver disease. Hepat Med.

[50] Moore AH (2019). Thrombocytopenia in cirrhosis: a review of pathophysiology and management options. Clin Liver Dis (Hoboken).

[51] Koruk M, Onuk MD, Akçay F (2002). Serum thrombopoietin levels in patients with chronic hepatitis and liver cirrhosis, and its relationship with circulating thrombocyte counts. Hepatogastroenterology.

[52] Terrault N, Chen YC, Izumi N (2018). Avatrombopag before procedures reduces need for platelet transfusion in patients with chronic liver disease and thrombocytopenia. Gastroenterology.

[53] Seeff LB, Everson GT, Morgan TR (2010). Complication rate of percutaneous liver biopsies among persons with advanced chronic liver disease in the HALT-C trial. Clin Gastroenterol Hepatol.

[54] Afdhal N, McHutchison J, Brown R (2008). Thrombocytopenia associated with chronic liver disease. J Hepatol.

[55] Massoud OI, Zein NN (2017). The effect of transjugular intrahepatic portosystemic shunt on platelet counts in patients with liver cirrhosis. Gastroenterol Hepatol (N Y).

[56] Afdhal NH, Giannini EG, Tayyab G (2012). Eltrombopag before procedures in patients with cirrhosis and thrombocytopenia. N Engl J Med.

[57] Hidaka H, Kurosaki M, Tanaka H (2019). Lusutrombopag reduces need for platelet transfusion in patients with thrombocytopenia undergoing invasive procedures. Clin Gastroenterol Hepatol.

[58] Tateishi R, Seike M, Kudo M (2019). A randomized controlled trial of lusutrombopag in Japanese patients with chronic liver disease undergoing radiofrequency ablation. J Gastroenterol.

[59] Moussa MM, Mowafy N (2013). Preoperative use of romiplostim in thrombocytopenic patients with chronic hepatitis C and liver cirrhosis. J Gastroenterol Hepatol.

[60] Liangpunsakul S, Ulmer BJ, Chalasani N (2003). Predictors and implications of severe hypersplenism in patients with cirrhosis. Am J Med Sci.

[61] Scheiner B, Semmler G, Maurer F (2020). Prevalence of and risk factors for anaemia in patients with advanced chronic liver disease. Liver Int.

[62] Piano S, Tonon M, Vettore E (2017). Incidence, predictors and outcomes of acute-on-chronic liver failure in outpatients with cirrhosis. J Hepatol.

